# Supplementary material for: Longitudinal protein profiling of blood during childhood into early adulthood
Source: Nat Commun. 2026 Apr 22;17:3700. doi: 10.1038/s41467-026-72095-3 (PMC13102979; doi:10.1038/s41467-026-72095-3)
Supplement: Supplementary file 1 — Supplementary Information [file 41467_2026_72095_MOESM1_ESM.pdf]

Supplementary Information for:

Longitudinal protein profiling of blood during childhood into early adulthood

Sofia Bergström[#1], Sophia Björkander[#*2], María Bueno Álvez[1], Simon Kebede Merid[2], Hanna Danielsson[3,4], Anna Bergström[5,6], Inger Kull[2,4], Anne-Sophie Merritt[5,6], Fredrik Edfors[1], Susanna Klevebro[2,4], Mathias Uhlén[1], Peter Nilsson[$1], Erik Melén[$2,4]

[#]These authors contributed equally
[$]These authors jointly supervised the work

1. Department of Protein Science, KTH Royal Institute of Technology, SciLifeLab, Stockholm, Sweden
2. Department of Clinical Science and Education, Södersjukhuset, Karolinska Institutet, Stockholm, Sweden
3. Department of Women's and Children's Health, Karolinska Institutet, Stockholm, Sweden
4. Sachs' Children and Youth Hospital, Södersjukhuset, Stockholm, Sweden
5. Institute of Environmental Medicine, Karolinska Institutet, Stockholm, Sweden
6. Centre for Occupational and Environmental Medicine, Region Stockholm, Stockholm, Sweden

*Corresponding author
Sophia Björkander, e-mail: sofia.bjorkander@ki.se

Supplementary Figures

Supplementary Figure 1.

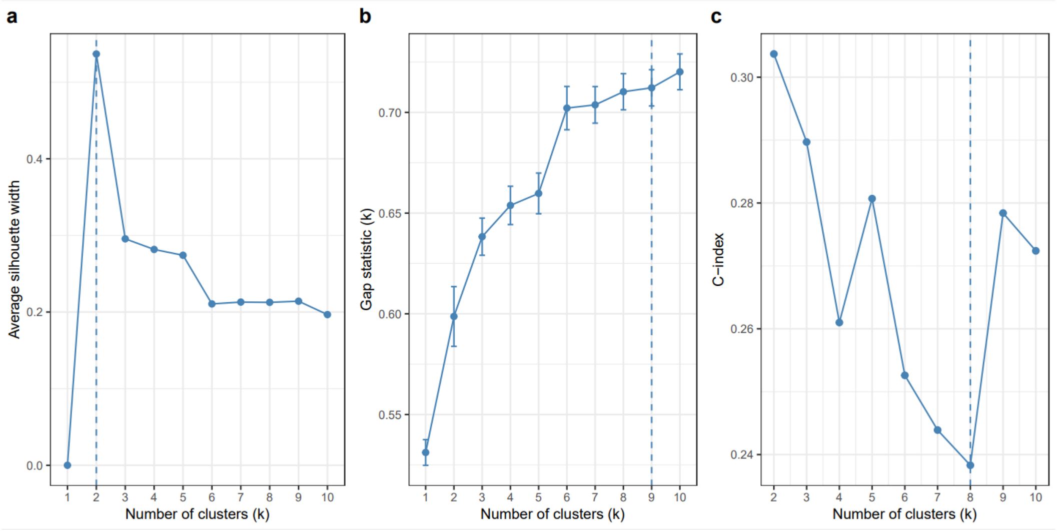

Figure S1: Assessment of optimal number of clusters.
Optimal number of clusters assessed by average Silhouette width (a), Gap statistic (b), and C-index (c).

Supplementary Figure 2.

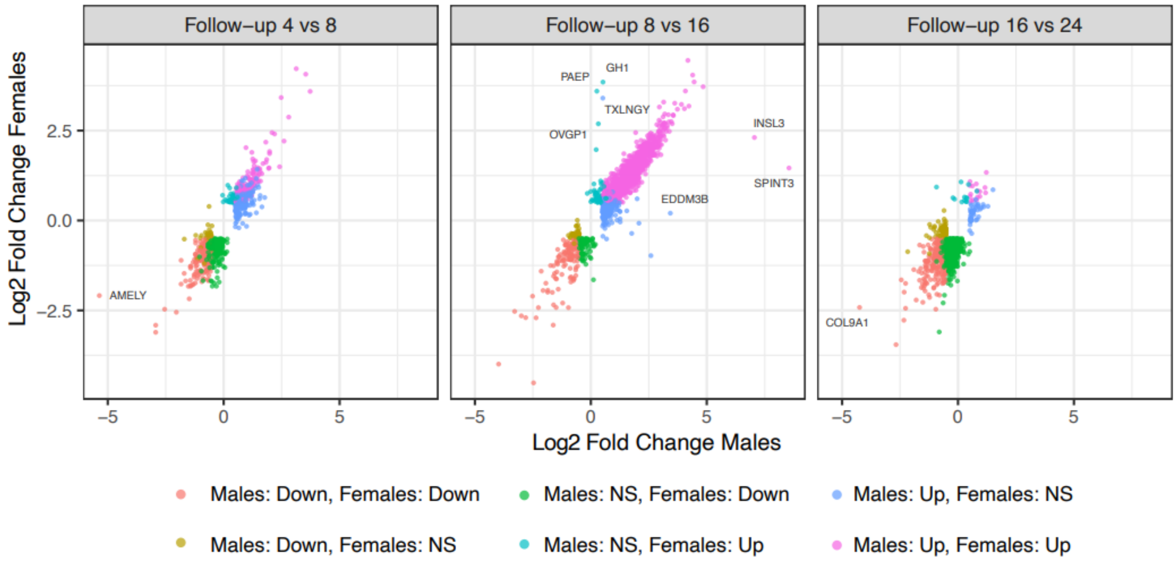

Figure S2: Correlations of log2 fold changes in females and males obtained from the comparisons of protein levels between consecutive follow-ups.
Proteins were first filtered on log2 fold change >0.5 and FDR p-value <0.05. Left: comparison follow-ups 4 and 8. Middle: comparison follow-ups 8 and 16. Right: comparison follow-ups 16 and 24. Log2 fold changes for females are displayed on the y-axis and Log2 fold change for males are displayed on the x-axis. NS=non-significant. Down=lower protein levels at the latter follow-up. Up=higher protein levels at the latter follow-up. The two-tailed

Wilcoxon signed-rank test corrected for multiple testing using the Benjamini-Hochberg procedure ($p<0.05$) and a log2 fold change was used to assign statistical significance.
