# Supplementary material for: Longitudinal protein profiling of blood during childhood into early adulthood
Source: Nat Commun. 2026 Apr 22;17:3700. doi: 10.1038/s41467-026-72095-3 (PMC13102979; doi:10.1038/s41467-026-72095-3)
Supplement: Supplementary file 2 — Description Of Additional Supplementary File [file 41467_2026_72095_MOESM2_ESM.pdf]

Description of Additional supplementary files

Supplementary Data 1. Comparison of protein levels between consecutive follow-ups.
Displays protein names, raw p-values, BH-corrected p-values, log2 fold change where negative log2 fold change corresponds to lower levels at the earlier follow-up, and cluster belonging. The two-tailed Wilcoxon signed-rank test corrected for multiple testing using the Benjamini-Hochberg procedure ($p<0.05$) and a log2 fold change was used to assign statistical significance.

Supplementary Data 2. Comparison of protein levels between consecutive follow-ups in females and males separated.
Displays protein names, raw p-values, BH-corrected p-values, log2 fold change where negative log2 fold change corresponds to lower levels at the earlier follow-up. The two-tailed Wilcoxon signed-rank test corrected for multiple testing using the Benjamini-Hochberg procedure ($p<0.05$) and a log2 fold change was used to assign statistical significance.

Supplementary Data 3. GO BP terms for clusters based on age-associated analysis.
Displays all significant GO BP terms for each cluster in Figure 3. For the overrepresentation analysis, a one-sided version of Fisher's exact test, corrected for multiple testing using the Benjamini-Hochberg procedure ($p<0.05$), was used to assign statistical significance.

Supplementary Data 4. Comparison of protein levels between females and males within each follow-up.
Displays protein names, raw p-values, BH-corrected p-values and log2 fold change where negative log2 fold change corresponds to higher levels in males. The two-tailed Wilcoxon rank sum test corrected for multiple testing using the Benjamini-Hochberg procedure ($p<0.05$) and a log2 fold change was used to assign statistical significance.

Supplementary Data 5. GO BP terms for proteins differing between males and females.
Displays all significant GO BP terms for proteins that significantly differ between the sexes at follow-up 16, follow-up 24 or follow-up 16 and 24. For the overrepresentation analysis, a one-sided version of Fisher's exact test, corrected for multiple testing using the Benjamini-Hochberg procedure ($p<0.05$), was used to assign statistical significance.

Supplementary Data 6. Proteins associated with body fat percentage (BF%) in linear regression analysis.
Displays the 22 proteins found to be significantly associated with body fat percentage, out of which 14 proteins remained significantly associated with sex after adjustments.
