# Supplementary material for: Longitudinal protein profiling of blood during childhood into early adulthood
Source: Nat Commun. 2026 Apr 22;17:3700. doi: 10.1038/s41467-026-72095-3 (PMC13102979; doi:10.1038/s41467-026-72095-3)
Supplement: Supplementary file 4 — Reporting summary [file 41467_2026_72095_MOESM4_ESM.pdf]

nature portfolio

Corresponding author(s): Sophia Björkander

Last updated by author(s): April 2nd, 2026

Reporting Summary

Nature Portfolio wishes to improve the reproducibility of the work that we publish. This form provides structure for consistency and transparency in reporting. For further information on Nature Portfolio policies, see our Editorial Policies and the Editorial Policy Checklist.

Statistics

For all statistical analyses, confirm that the following items are present in the figure legend, table legend, main text, or Methods section.

n/a	Confirmed	
☐	☒	The exact sample size (*n*) for each experimental group/condition, given as a discrete number and unit of measurement
☐	☒	A statement on whether measurements were taken from distinct samples or whether the same sample was measured repeatedly
☐	☒	The statistical test(s) used AND whether they are one- or two-sided<br>*Only common tests should be described solely by name; describe more complex techniques in the Methods section.*
☐	☒	A description of all covariates tested
☐	☒	A description of any assumptions or corrections, such as tests of normality and adjustment for multiple comparisons
☐	☒	A full description of the statistical parameters including central tendency (e.g. means) or other basic estimates (e.g. regression coefficient) AND variation (e.g. standard deviation) or associated estimates of uncertainty (e.g. confidence intervals)
☐	☒	For null hypothesis testing, the test statistic (e.g. *F*, *t*, *r*) with confidence intervals, effect sizes, degrees of freedom and *P* value noted<br>*Give P values as exact values whenever suitable.*
☒	☐	For Bayesian analysis, information on the choice of priors and Markov chain Monte Carlo settings
☐	☒	For hierarchical and complex designs, identification of the appropriate level for tests and full reporting of outcomes
☐	☒	Estimates of effect sizes (e.g. Cohen's *d*, Pearson's *r*), indicating how they were calculated

Our web collection on statistics for biologists contains articles on many of the points above.

Software and code

Policy information about availability of computer code

Data collection	Proteomics measurement were perfomed using the Olink Explore HT (OlinkTM) platform by a combination of Proximity Extension Assay and next-generation sequencing (www.proteinatlas.org). Questionnaire data was obtained by parent-answered (follow-ups 4 and 8) or self-answered (follow-ups 16 and 24) questionnaires. Clinical variables (height, weight, blood cell counts) were obtained during clinical examinations conducted by research nurses and doctors.
Data analysis	Demographic and subject background variables were analyzed using STATA SE (version 16). All other analysis was performed using R Software (version 4.5.1) and the following packages: OlinkAnalyze package (version 4.3.1), factoextra::fviz_pca_ind, version 1.0.7, UpSetR:: upset, version 1.4.0, factoextra::fviz_nbclust(method = "gap_stat" or method = "silhouette"), version 1.0.7, NbClust:: NbClust(index = "cindex", version 3.0.1, fcp::hclustCBI and fcp::clusterboot, version 2.2-13, clusterProfiler::enrichGO, version 4.16.0, org.Hs.eg.db database (version 3.21.0), lme4:: lme, version 1.1.37, lmerTest, version 3.1.3. Codes are publicly available at GitHub (https://github.com/sofiabergstrom/childhood-protein-profiling) and Zenodo (https://zenodo.org/account/settings/github/repository/sofiabergstrom/childhood-protein-profiling, DOI: https://doi.org/10.5281/zenodo.19099156).

For manuscripts utilizing custom algorithms or software that are central to the research but not yet described in published literature, software must be made available to editors and reviewers. We strongly encourage code deposition in a community repository (e.g. GitHub). See the Nature Portfolio guidelines for submitting code & software for further information.

Data

Policy information about availability of data

All manuscripts must include a data availability statement. This statement should provide the following information, where applicable:

- Accession codes, unique identifiers, or web links for publicly available datasets
- A description of any restrictions on data availability
- For clinical datasets or third party data, please ensure that the statement adheres to our policy

All data associated with this study are available within this paper, its supplementary information or in the Source data, or may be obtained upon request from the corresponding author. Due to consent and legal constraints, the individual-level data cannot be deposited openly. Access to individual-level data can be granted for both non-commercial validation or collaborative purposes and upon request to the corresponding author. A request should contain the following: the name of PI and host organization, contact details, the scientific purpose of the data access request, the commitment to inform when the data has been used in a publication, the commitment not to host or share the data outside the requesting organization, and a statement of non-commercial use of data. Data access requests will be responded to within four weeks unless ethical constraints require new ethical permits, and the data will be available for a mutually decided timeframe. Source data are provided with this paper.

Research involving human participants, their data, or biological material

Policy information about studies with human participants or human data. See also policy information about sex, gender (identity/presentation), and sexual orientation and race, ethnicity and racism.

Reporting on sex and gender	In the study cohort, sex was determined by birth data from the Swedish National Board of Health and Welfare ("Socialstyrelsen" in Swedish). In the current study, sex was included as a selection criterion (equal numbers of female participants and male participants in random sampling). Distribution of clinical variables are displayed stratified for sex in Table 1.
Reporting on race, ethnicity, or other socially relevant groupings	The study included Swedish participants, >95% of white descent. No variables related to social grouping was used in the analysis.
Population characteristics	Population characteristics are described in Table 1.
Recruitment	100 subjects (50 male participants, 50 female participants) were randomly selected from the BAMSE cohort. All children who were born in four pre-defined municipalities in Stockholm between 1994-1996 were invited to participate (n=7221) in BAMSE, out of which 5488 children were eligible (exclusion: planned move, insufficient knowledge in the Swedish language, serious illness of the child or siblings already included). Out of all eligible subjects, 4089 agreed to participate (>95% white participants).
Ethics oversight	This study was conducted in accordance with the Declaration of Helsinki and was approved by the Swedish Ethical Review Authority and the Regional Ethics Review Board in Stockholm (Approval 2016/1380-31/2). All participants and/or their legal guardian gave written informed consent to participate in the study.

Note that full information on the approval of the study protocol must also be provided in the manuscript.

Field-specific reporting

Please select the one below that is the best fit for your research. If you are not sure, read the appropriate sections before making your selection.

☒ Life sciences ☐ Behavioural & social sciences ☐ Ecological, evolutionary & environmental sciences

For a reference copy of the document with all sections, see nature.com/documents/nr-reporting-summary-flat.pdf

Life sciences study design

All studies must disclose on these points even when the disclosure is negative.

Sample size	The data presented here is a subset of the data generated within the Human Disease Blood Atlas project as a part of the Human Protein Atlas (www.proteinatlas.org). The sample-size was determined based on availability of resources within the Human Disease Atlas project.
Data exclusions	All proteins from dilution block 8, corresponding to the most abundant proteins (n = 68, dilution 1:100 000), were excluded from the analyses due to technical issues following the recommendation from the provider. Three individuals had sample quality control warnings for 270-1204 proteins each, and those individuals were excluded from the dataset to get complete longitudinal data, no values were imputed.
Replication	Finding were validated in two external published datasets, Niu et al. (2025) and Liu et al. (2017), by mimicking the statistical methods used in these studies. Overall replication success was around 70%.
Randomization	Samples were randomized to plates within the same batch, longitudinal samples from the same individual were analyzed on the same plate.

Blinding	Blinding was not relevant for this study, as no treatment groups were compared and no non-objective interpretation of data was conducted.

Reporting for specific materials, systems and methods

We require information from authors about some types of materials, experimental systems and methods used in many studies. Here, indicate whether each material, system or method listed is relevant to your study. If you are not sure if a list item applies to your research, read the appropriate section before selecting a response.

Materials & experimental systems

n/a	Involved in the study	
☐	☒	Antibodies
☒	☐	Eukaryotic cell lines
☒	☐	Palaeontology and archaeology
☒	☐	Animals and other organisms
☐	☒	Clinical data
☒	☐	Dual use research of concern
☒	☐	Plants

Methods

n/a	Involved in the study	
☒	☐	ChIP-seq
☒	☐	Flow cytometry
☒	☐	MRI-based neuroimaging

Antibodies

Antibodies used	As provided by Olink
Validation	As validated by the Human Protein Atlas

Clinical data

Policy information about clinical studies

All manuscripts should comply with the ICMJE guidelines for publication of clinical research and a completed CONSORT checklist must be included with all submissions.

Clinical trial registration	NA
Study protocol	NA
Data collection	NA
Outcomes	NA

Plants

Seed stocks	*Report on the source of all seed stocks or other plant material used. If applicable, state the seed stock centre and catalogue number. If plant specimens were collected from the field, describe the collection location, date and sampling procedures.*
Novel plant genotypes	*Describe the methods by which all novel plant genotypes were produced. This includes those generated by transgenic approaches, gene editing, chemical/radiation-based mutagenesis and hybridization. For transgenic lines, describe the transformation method, the number of independent lines analyzed and the generation upon which experiments were performed. For gene-edited lines, describe the editor used, the endogenous sequence targeted for editing, the targeting guide RNA sequence (if applicable) and how the editor was applied.*
Authentication	*Describe any authentication procedures for each seed stock used or novel genotype generated. Describe any experiments used to assess the effect of a mutation and, where applicable, how potential secondary effects (e.g. second site T-DNA insertions, mosiacism, off-target gene editing) were examined.*
